# Epidemiology of cardiovascular diseases related admissions in a referral hospital in the South West region of Cameroon: A cross-sectional study in sub-Saharan Africa

**DOI:** 10.1371/journal.pone.0226644

**Published:** 2019-12-19

**Authors:** Clovis Nkoke, Ahmadou Musa Jingi, Christelle Makoge, Denis Teuwafeu, Cyrille Nkouonlack, Anastase Dzudie

**Affiliations:** 1 Buea Regional Hospital, Buea, Cameroon; 2 Clinical Research Education, Networking and Consultancy, Douala, Cameroon; 3 Faculty of Medicine and Biomedical Sciences, University of Yaounde, Yaounde, Cameroon; 4 Faculty of Health Sciences, University of Buea, Buea, Cameroon; 5 Douala General Hospital, Douala, Cameroon; Azienda Ospedaliero Universitaria Careggi, ITALY

## Abstract

**Background:**

Sub-Saharan Africa (SSA) is experiencing an epidemic of cardiovascular diseases (CVD) as a result of a rapid epidemiological transition. Little is known about the admission for CVD and outcome in rural and semi-urban settings in Cameroon in this era of epidemiological transition. The aim of this study was to determine the frequency and the pattern of CVD admissions in the South West region of Cameroon.

**Methods:**

This retrospective descriptive study included all adult patients admitted for CVD in the medical unit of the Buea Regional Hospital between Jan 2016 and December 2017.

**Results:**

Out of the 3140 patients admitted, 499(15.9%) had CVD. There were 304(60.9%) females. The mean age was 58.7±16.2 years. There was no age difference between men and women (59.7 years vs 58.1years, p = 0.29). The most commonly affected age group was those aged 50–59 years (22%). Heart failure (38.5%), stroke (33.3%) and uncontrolled hypertension (22.4%) were the most prevalent CVDs. The length of hospital stay ranged from 1 to 37 days with a median length of hospital stay of 7 days. In-hospital case fatality was 78(15.8%). Mortality was higher in women compared to men (9% vs 7%, p = 0.43). The case fatality for stroke was higher compared to case fatality for heart failure (21.7% vs 16.7%, p = 0.23).

**Conclusion:**

CVDs are a common cause of hospital admission in this semi-urban setting, dominated by heart failure. Women were disproportionately affected and it was associated with high mortality. Prevention, early detection and management of risk factors for cardiovascular disease are imperative given the growing burden of CVD in SSA to reduce CVD morbidity and mortality.

## Introduction

Sub-Saharan Africa (SSA) is facing a double burden of infectious and non-communicable diseases including cardiovascular disease (CVD). Cardiovascular diseases (CVD) are a major public health problem worldwide. It is the leading cause of death worldwide with about 80% of the deaths occurring in low and middle-income countries including SSA [1].

Sub-Saharan Africa (SSA) is experiencing an epidemic of CVD as a result of the rapid epidemiological transition with urbanization and adoption of western lifestyles [2, 3]. As a result of this epidemiological transition, the incidence of CVD in SSA has been on a rise in the last few years [4]. This places an additional burden on the health care systems which are already overwhelmed by infectious diseases and limited resources. In 2015, about 1.2 million people died from CVD in Africa [5] and the CVD burden of SSA is projected to double by 2030, predominantly driven by increased rates of hypertension, smoking, and obesity [6]. It is postulated that CVD is set to overcome infectious diseases in developing countries to become the most common causes of death [7, 8]. A study in Ghana, on the trend of CVD admission, showed an increase in the percentage of CVD admissions from 4.6% to 8.2%, representing a 78% increase over a decade [9].

There is a paucity of data regarding the burden of CVD admissions in Cameroon in this era of epidemiological transition which limits the formulation of data-driven national policies. Also there are no morbidity and mortality registries for CVD in Cameroon. The goal of this study was to determine the frequency and pattern of CVD admission in the Buea Regional Hospital, South West region of Cameroon. The study could support policy makers to develop strategies to fight CVD.

## Methodology

### Study setting and design

This was a retrospective study of patients with a diagnosis of CVD admitted to the medical wards of the Buea Regional Hospital between January 2016 and December 2017. This is a secondary level Hospital and serves as one of the two main referral centers in the region, with a bed capacity of about 111 beds, and a catchment population of about 200,000 inhabitants. The Hospital which is a public hospital also serves as one of the teaching hospitals of the University of Buea. Buea is a semi-urban setting, the headquarters of the South West region of Cameroon. The main economic activity in the region is agriculture. The hospital receives referrals from other health facilities in the region.

### Data collection

The data was collected from the ward admission and discharge register. Details obtained included age, sex, a final diagnosis of CVD, duration of hospitalization and outcome. All cases were diagnosed by a physician using clinical features and investigations. Patients with an unclear diagnosis of cardiovascular disease or incomplete data were excluded.

### Ethical statement

The study was approved by the administrative authorities of the hospital acting as the local ethics committee. We carried out this work in accordance with the declarations of Helsinki. We report this work following the preferred standard in reporting observational studies in epidemiology (STROBE). No informed consent was required since it was a retrospective review the using admission and discharge register.

### Sample size and statistical analysis

A convenient sample of all eligible patients was considered for this study. The data collected were analyzed using SPSS version 20. Data were expressed as frequency and percentages for discrete variables, and as means (with standard deviation and confidence interval of the means) for continuous variables. We have presented the distribution of CVDs according to sex and age group. We compared the proportions using the Chi-squared test or Fischer exact test where appropriate. We have compared mean values using the Student t-test. We considered a p-value <0.05 to be statistically significant for the observed differences or associations.

## Results

**Fig 1:**
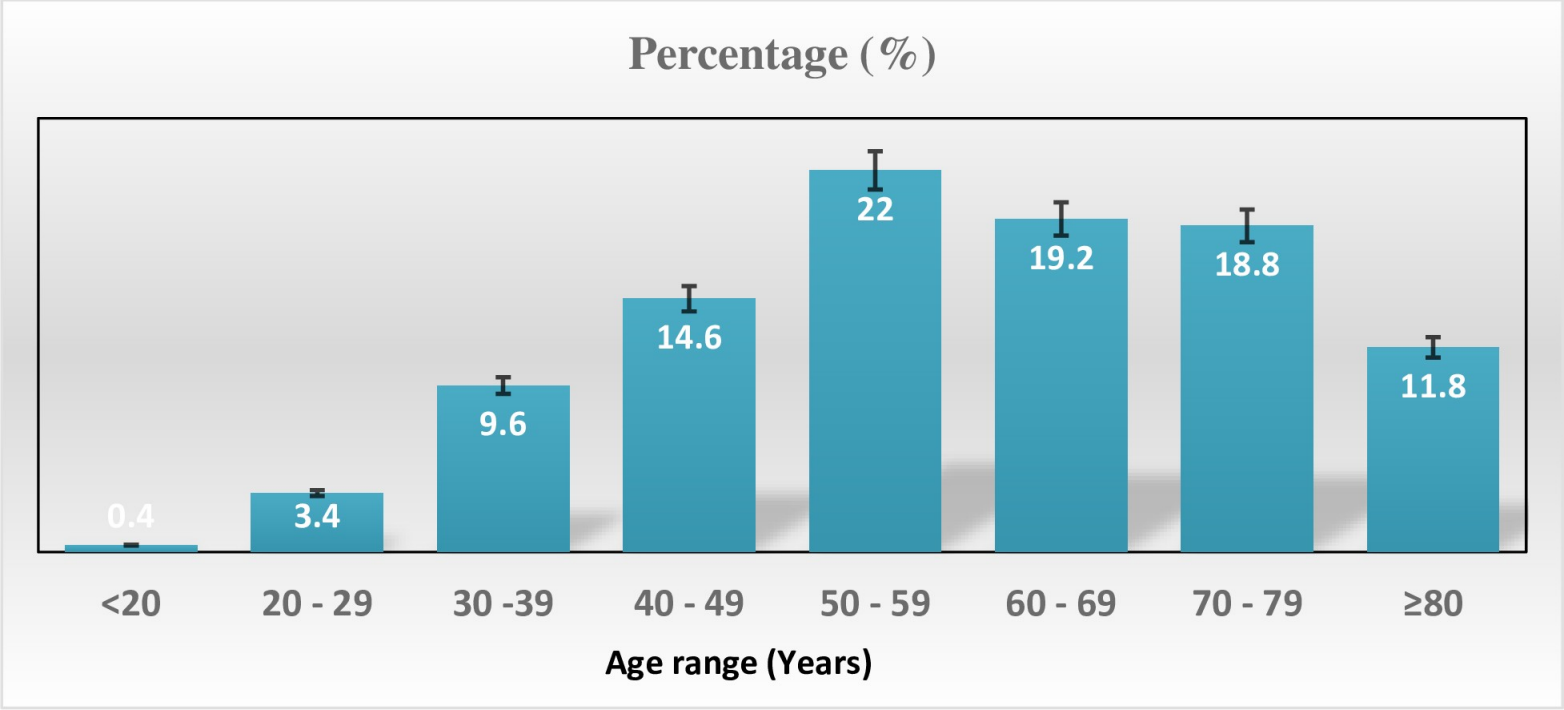
Age distribution of patients.

During the two year study period, a total of 3140 patients were admitted and 499(15.9%) were admitted for CVD. There were 304(60.9%) females. The mean age was 58.7±16.2 years. There was no age difference between men and women (59.7 years vs 58.1 years, p = 0.29). The most commonly affected age group (Fig 1) was those aged 50–59 years (22%). Heart failure (38.5%), stroke (33.3%), and uncontrolled hypertension (22.4%) were the most common CVDs. The distribution of CVDs according to sex is shown in Table 1. The proportion of patients admitted for stroke was significantly higher in men, while uncontrolled hypertension was significantly more common in females. The distribution of CVDs according to the age group is shown in Table 2. Stroke and heart failure admissions increased with increasing age. Uncontrolled hypertension was more common between 40 and 60 years. Poor outcome (death) and mean duration of hospitalization are shown in Table 3. The highest burden of death was seen in those with heart failure and stroke. The length of hospital stay ranged from 1 to 37 days with a median length of hospital stay of 7 days. In-hospital case fatality was 78(15.8%). Mortality was higher in women compared to men (9% vs 7%, p = 0.43). The case-fatality for stroke was higher compared to case-fatality for heart failure (21.7% vs 16.7%, p = 0.23).

**Table 1:** Distribution of CVD by sex.

Cardiovascular disease	Total(N = 499)	Sex		*p* value
		Male(n = 195)	Female(n = 304)	
Stroke	166(33.3)	78(40)	88(29)	**0.011**
Heart failure	192(38.5)	76(39)	116(38.2)	0.858
Uncontrolled hypertension	112(22.4)	32(16.4)	80(26.3)	**0.0098**
Acute myocardial infarction	4(0.8)	3(1.5)	1(0.3)	0.135
Venous thromboembolism	23(4.5)	8(4.1)	15(4.9)	0.677
Pericardial disease	3(0.6)	1(0.5)	2(0.7)	0.782
Arrhythmias	5(1)	2(1)	3(1)	1

**Table 2:** Distribution of CVD by age distribution.

Cardiovascular Disease	Age range (Years)						
	<30	30–39	40–49	50–59	60–69	≥ 70	Total
Stroke	1(0.6)	11(6.6)	27(16.3)	36(21.7)	34(20.5)	57(34.3)	166(100)
Heart failure	5(2.6)	13(6.8)	15(7.8)	44(22.9)	38(19.8)	77(40.1)	192(100)
Uncontrolled hypertension	11(9.8)	20(17.9)	26(23.2)	26(23.2)	15(13.4)	14(12.5)	112(100)
Acute myocardial infarction	0(0)	0(0)	0(0)	1(25)	2(50)	1(25)	4(100)
Venous thromboembolism	1(4.3)	5(21.7)	5(21.7)	6(26.1)	4(17.4)	2(8.7)	23(100)
Pericardial disease	1(33.3)	0(0)	0(0)	0(0)	2(66.7)	0(0)	3(100)
Arrhythmias	0(0)	0(0)	0(0)	0(0)	2(40)	3(60)	5(100)

**Table 3:** Poor outcome and mean hospital stay.

	Died in Hospital		Duration in Hospital	
Cardiovascular disease	Frequency	%	Mean (SD)	95% CI of Mean
Stroke	36/166	21.7	8 (5.1)	7.3–8.8
Heart failure	32/192	16.7	7.9 (5.3)	7.2–8.7
Uncontrolled hypertension	8/112	7.1	5.2 (3.7)	4.5–5.9
Acute myocardial infarction	0/4	0	5 (2)	1.8–8.2
Venous thromboembolism	2/23	8.2	10.9 (6.2)	8.2–13.5
Pericardial disease	2/3	66.7	13.3 (9.3)	-9.6–36.4
Arrhythmias	1/5	20	5.6 (7.8)	-4.1–15.3

## Discussion

The aim of this study was to report on the epidemiology of CVD admissions in a semi-urban setting in Cameroon. We found that CVD accounted for 15.9% of admissions in the medical ward. Women were significantly more affected than men; the most common CVDs were heart failure, stroke and uncontrolled hypertension. In-hospital case-fatality was 15.8%.

The epidemic of CVD associated with lifestyle and epidemiological transitions occurring all over the world is increasingly being recognized. Cardiovascular disease accounts for one-third of global deaths and it is a leading contributor to the global burden of disease. This is the first study describing the epidemiology of CVD admissions in the South West region of Cameroon, a semi- urban setting in this era of epidemiological transition. The incidence of CVD admission in this study which was about 16% was similar to the admission for CVD reported by *Osuji et al* in Nigeria [10]. This was however higher than the admission for CVD reported in the Northern part of Cameroon (9.9%) [11]. It was also higher than that reported by previous studies that showed that CVD accounted for approximately 7%-9% of admissions among adults in medical wards in Africa [12]. But more recent reports have shown prevalence of up to 20.1% in Nigeria [13]. This increasing prevalence of CVD admission in SSA can be attributed to the deteriorating cardiovascular risk factors profile of populations in SSA as a result of epidemiological transition with a rising prevalence of hypertension, diabetes, obesity and overweight [4,6]. A study in Ghana demonstrated an increasing percentage of CVD admissions from 4.6% to 8.2%, representing a 78% increase over a decade [9]. In Cameroon, stroke admission in a major referral hospital increased from 2.5% in 1999–2000 to 13.1% in 2011–2012 [14].

The incidence of CVD increases with increasing age. The mean age in our study which was 58.7 years was close to that reported by *Osuji et al* in Nigeria [15]. It was however higher than that reported by other authors in other African countries [16, 17]. The majority of patients admitted for CVD were between 50–69 years, an observation that was similar to that reported in Nigeria where the majority of patients admitted for CVD were aged 51–74 years [13]. The greater incidence of CVD in patients over 50 years of age is consistent with the overall trend of CVD which increases with increasing age [18]. But studies have shown that CVD occurs at a younger age in low and middle-income countries including SSA, affecting the economically active proportion of the population [19]. This will affect the economic productivity of these countries. The distribution of cardiovascular disease admissions by sex in Africa has been inconsistent. Overall, more women (60%) were admitted for CVD compared to men in our study. This is in contrast with reports from Nigeria where the proportion of men (60%) admitted for CVD was higher [15]. In Ghana, there was a nearly equal distribution of CVD admission by sex [9]. The risk of CVD in women increases after menopause. This may explain the higher prevalence of CVD admission in women in this study [20–22]. This higher prevalence of CVD admission in women in this study may also be attributed to differences in access to healthcare.

In our study, heart failure constituted the first cause of CVD admission with 38.5%. This was a similar finding in Ghana where it represented 80% of CVD admissions [9]. Stroke was the second cause of CVD admission in our study. On the contrary, stroke was the first CVD necessitating admission in medical wards in some studies from Nigeria [15, 23]. Heart failure has emerged as the most common primary diagnosis for patients admitted to the hospital with suspected cardiac disease in SSA [24].

Uncontrolled hypertension was the third cause of CVD admission in our study. Hypertension is by far the largest contributor to the CVD burden worldwide. A recent nationwide survey in the Cameroonian adult population reported a prevalence of hypertension of 29.2% [25]. Hypertension was the single most common etiological risk factor reported in studies on heart failure and stroke in Cameroon [14, 26]. This high prevalence of hypertension is associated with low awareness and poor control [25, 27]. Awareness of CVD and its risk factors by the general population is low. In a population-based survey in Cameroon, *Aminde et al* reported that more than half of the participants had a poor knowledge on CVD and its risk factors [28]. Also, only one quarter of the participants could correctly identify different types of CVD. In sub-Saharan Africa, the prevention, detection, management and control of hypertension should now be regarded as a high priority.

The in-hospital case fatality for CVD in our study was 15.8%. This was lower than that reported in Nigeria where the authors reported a mortality of 32%. But similar to study, mortality was highest in patients admitted for stroke [15]. According to the WHO, over three quarters of CVD death occur in low and middle income countries [1].

### Limitations

This study may underestimate the occurrence of cardiovascular diseases in the population. Patients with severe disease might have died at home or before reaching the hospital. These patients would not be reported in the hospitalization registries, thus would not be captured in this study. However, this study provides useful information on the epidemiology of cardiovascular diseases in this region, in an era of epidemiological transition.

## Conclusion

Cardiovascular disease accounts for a significant proportion of medical admission. There was a female predisposition to stroke and uncontrolled hypertension related admissions. Prevention, early detection and management of risk conditions should now be regarded as high a priority given the growing burden.

## Supporting information

S1 Database (XLSX)
